# A Case of Cecal Mixed Neuroendocrine–Non-Neuroendocrine Neoplasm Requiring Adjuvant Chemotherapy Based on Comprehensive Pathological Assessment

**DOI:** 10.70352/scrj.cr.26-0444

**Published:** 2026-07-14

**Authors:** Ryoma Yokoi, Keita Matsumoto, Chika Mizutani, Masahiro Fukada, Yuta Sato, Itaru Yasufuku, Chika Takao, Ryuichi Asai, Jesse Yu Tajima, Yoshihiro Tanaka, Tatsuhiko Miyazaki, Nobuhisa Matsuhashi

**Affiliations:** 1 Department of Gastroenterological Surgery and Pediatric Surgery, Gifu University Graduate School of Medicine, Gifu, Gifu, Japan; 2 Division of Pathology, Gifu University Hospital, Gifu, Gifu, Japan

**Keywords:** mixed neuroendocrine–non-neuroendocrine neoplasm (MiNEN), mixed adenoneuroendocrine carcinoma (MANEC), colorectal cancer, neuroendocrine carcinoma, adjuvant chemotherapy

## Abstract

**INTRODUCTION:**

Colorectal mixed neuroendocrine–non-neuroendocrine neoplasms (MiNENs) are rare and aggressive tumors characterized by marked intratumoral heterogeneity, often making preoperative diagnosis difficult. Consequently, definitive diagnosis is frequently established only after surgical resection, and postoperative pathological findings may influence subsequent treatment strategies.

**CASE PRESENTATION:**

A 66-year-old man presented with bloody stool. Colonoscopy revealed a cecal tumor, and biopsy demonstrated moderately differentiated adenocarcinoma. Laparoscopic ileocecal resection with D3 lymph node dissection was performed. Histopathological examination of the resected specimen revealed a collision-type MiNEN composed of neuroendocrine carcinoma (NEC) (60%) and adenocarcinoma (40%). The NEC component showed lymphovascular invasion, deep mesenteric invasion, and a Ki-67 index of approximately 50%–60%. Although regional lymph node metastasis involved only the adenocarcinoma component, comprehensive pathological assessment suggested biologically aggressive behavior of the NEC component, raising concern for potential systemic dissemination. Accordingly, NEC-oriented adjuvant chemotherapy with carboplatin plus etoposide was administered after curative resection. The patient remained recurrence-free until death from other causes 2 years after surgery.

**CONCLUSIONS:**

Colorectal MiNEN poses substantial diagnostic challenges because biopsy specimens may not adequately represent both tumor components. Comprehensive pathological assessment of the resected specimen is important for identifying the aggressive component most likely to influence prognosis and for optimizing postoperative therapeutic strategy.

## Abbreviations

CD56
: cluster of differentiation 56
MANEC
: mixed adenoneuroendocrine carcinoma
MiNEN
: mixed neuroendocrine–non-neuroendocrine neoplasm
NEC
: neuroendocrine carcinoma

## INTRODUCTION

MiNENs are rare malignant tumors defined by the coexistence of both neuroendocrine and non-neuroendocrine components, each accounting for at least 30% of the tumor.^1,2)^ Although MiNENs can arise throughout the gastrointestinal tract, colorectal involvement is relatively uncommon.^3)^ In colorectal MiNENs, the non-neuroendocrine component is most commonly adenocarcinoma, whereas the neuroendocrine component typically corresponds to poorly differentiated NEC. Such tumors were previously designated as MANECs in the 2010 World Health Organization (WHO) classification. The term MiNEN was subsequently introduced to encompass a broader spectrum of neuroendocrine and non-neuroendocrine components beyond adenocarcinoma and NEC.^1,2)^ High-grade colorectal MiNENs composed of adenocarcinoma and NEC are associated with aggressive biological behavior and poor prognosis comparable to that of pure NEC.^3–7)^ Because even a relatively small NEC component may adversely influence outcomes, treatment is generally directed toward the most aggressive tumor component, most commonly the NEC component, although optimal management remains controversial.^3,8–10)^ However, preoperative diagnosis is often difficult due to marked intratumoral heterogeneity. Consequently, many cases are diagnosed only after surgical resection, and postoperative pathological findings may influence subsequent treatment strategies.^3)^

Here, we report a case of cecal collision-type MiNEN that was diagnosed after upfront surgical resection and successfully managed with adjuvant platinum-based chemotherapy based on comprehensive pathological assessment.

## CASE PRESENTATION

**Fig. 1:**
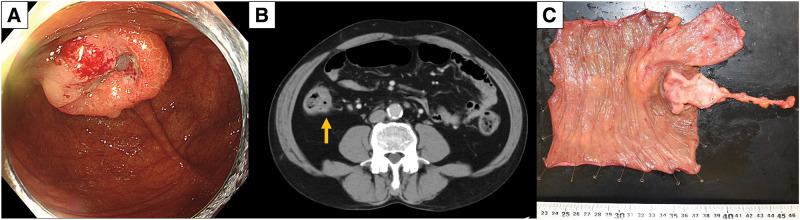
Preoperative examinations and macroscopic findings of the cecal tumor. (**A**) Colonoscopy revealed a type 2 ulcerative tumor in the cecum. (**B**) Contrast-enhanced abdominal CT demonstrated focal wall thickening of the cecum accompanied by regional lymphadenopathy (arrow). (**C**) The resected specimen showed an irregular ulcerative tumor arising from the appendiceal orifice of the cecum.

A 66-year-old man with a medical history of myocardial infarction and cerebral infarction, and comorbidities including diabetes mellitus, chronic kidney disease, and pulmonary emphysema, presented with bloody stool. Colonoscopy revealed a type 2 ulcerative tumor in the cecum (**Fig. 1A**), and biopsy demonstrated moderately differentiated adenocarcinoma. Contrast-enhanced CT showed cecal wall thickening with regional lymphadenopathy, without distant metastasis (**Fig. 1B**). Tumor markers were within normal limits. He was diagnosed with advanced cecal cancer (cT2N1bM0, cStage IIIA according to the TNM Classification of Malignant Tumors, 8th Edition). Laparoscopic ileocecal resection with D3 lymph node dissection was performed. The postoperative course was uneventful.

**Fig. 2:**
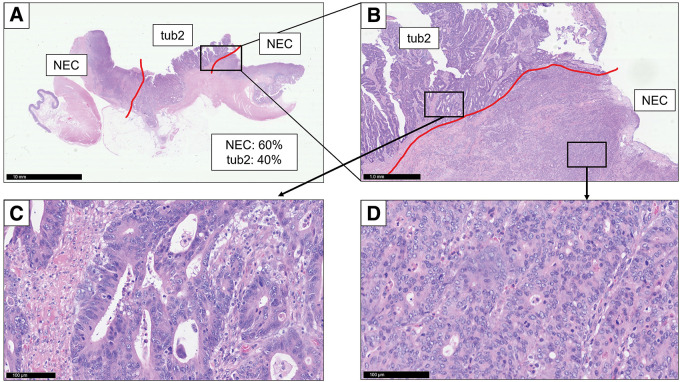
Histopathological findings of the cecal tumor on hematoxylin and eosin staining. Histopathological examination revealed 2 distinct malignant components: an NEC component and a moderately differentiated adenocarcinoma (tub2) component, forming a collision tumor. The boundary between the 2 components is indicated by red lines. (**A**) Low-power view showing the overall architecture of the tumor with spatially distinct NEC and adenocarcinoma components. (**B**) Intermediate-power view demonstrating a sharp interface between the 2 components. (**C**) High-power view of the moderately differentiated adenocarcinoma component. (**D**) High-power view of the NEC component, composed of relatively uniform tumor cells with scant cytoplasm and a high nuclear-to-cytoplasmic ratio. NEC, neuroendocrine carcinoma

**Fig. 3:**
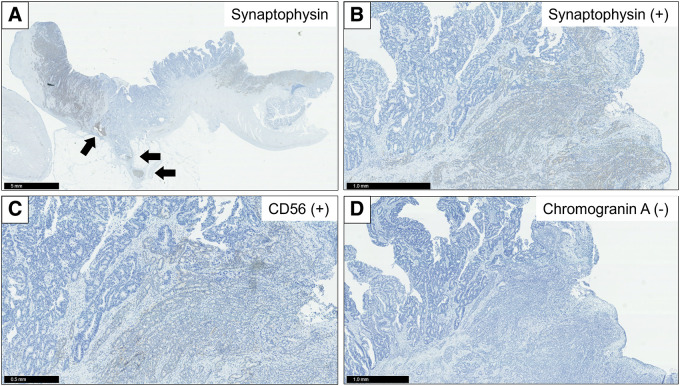
Immunohistochemical findings of the cecal tumor. Immunohistochemical analysis demonstrated that the NEC component was positive for synaptophysin (**A**, **B**) and CD56 (**C**), and negative for chromogranin A (**D**). In addition, the NEC component showed deep invasion into the mesentery (arrows), indicating aggressive local behavior. NEC, neuroendocrine carcinoma

The resected specimen revealed an irregular ulcerative tumor arising from the appendiceal orifice of the cecum (**Fig. 1C**). Histopathological examination revealed a MiNEN composed of NEC (60%) and adenocarcinoma (40%) with a collision-type pattern (**Fig. 2A**–**2D**). The NEC component was composed of relatively uniform tumor cells with scant cytoplasm, finely granular chromatin, and frequent mitotic figures (approximately 30 mitoses per 10 high-power fields). A delicate fibrovascular stroma was present throughout the lesion. According to the WHO classification, this component was diagnosed as small-cell NEC. Immunohistochemical analysis showed that the NEC component was positive for synaptophysin and CD56 and negative for chromogranin A (**Fig. 3A**–**3D**), with a Ki-67 index of approximately 50%–60%. One regional lymph node contained metastatic adenocarcinoma, and lymphovascular invasion was observed in both components. The NEC component also demonstrated deep invasion into the mesentery (**Fig. 3A**). The final pathological stage was pT3N1aM0, pStage IIIB according to the TNM Classification of Malignant Tumors, 8th Edition.

Given the aggressive NEC component, adjuvant chemotherapy with carboplatin plus etoposide was administered for 4 courses. The patient died of other causes 2 years after surgery without recurrence.

## DISCUSSION

Colorectal MiNENs are rare tumors characterized by marked intratumoral heterogeneity, which poses a major obstacle to accurate preoperative diagnosis. Biopsy specimens often fail to adequately represent both tumor components because of sampling limitations and diagnostic thresholds, resulting in a reported diagnostic accuracy of only approximately one third for MiNEN.^3,8,11,12)^ In the present case, preoperative biopsy demonstrated only moderately differentiated adenocarcinoma, whereas the resected specimen revealed a collision-type MiNEN composed predominantly of NEC. Collision-type MiNEN consists of 2 histologically distinct tumor components that coexist adjacently without histological intermingling, whereas composite-type MiNEN shows intimate intermingling of the 2 components.^8)^ In such collision-type tumors, superficial biopsy specimens may sample only the adenocarcinoma component, making preoperative diagnosis particularly difficult. In previously reported cases of colorectal collision-type MiNEN, several clinicopathological features have been consistently observed (**Table 1**).^13–24)^ These tumors predominantly arose in the right-sided colon, although some involved the rectum or anal canal. Only 1 of 13 reported cases was diagnosed as MiNEN preoperatively. Many cases were already locally advanced at diagnosis, with frequent nodal involvement and lymphovascular invasion, reflecting aggressive disease behavior. Distant recurrence—particularly hematogenous metastasis to the liver or lung—was commonly observed even after apparently curative resection, underscoring the systemic nature of colorectal MiNEN.

**Table 1:** Reported cases of colorectal collision-type MiNEN

Case no.	Author	Year	Age	Sex	Location	Preoperative diagnosis based on biopsy	Treatment	NE component (%)	Non-NE component (%)	Ki-67 (%)	T	N (component)	Lymphovascular invasion, component	Postoperative therapy	Recurrence (months after treatment, component)	Prognosis (months after treatment)
1	Kusakabe et al.^13)^	2014	71	M	Transverse	tub1	RHC	NEC (>30)	tub1 (>30)	25	3	1 (NEC + tub1)	N/D	No	Liver, lung (8, unknown)	Alive (13)
2	Minaya-Bravo et al.^14)^	2015	66	M	Transverse	tub	Subtotal colectomy	NEC (70)	tub2 (30)	20	3	1b (NEC)	(+) N/D	No	Retroperitoneal (36, NEC + tub2)	Alive (>36)
3	Morais et al.^15)^	2016	64	M	Ascending	Unknown due to insufficient material	RHC	NEC (30)	tub2 (70)	>20	3	1a (N/D)	N/D	FOLFOX	Liver (6, unknown)	Death (7)
4	Norose et al.^16)^	2017	54	M	Rectum	NEC, UC	Anterior resection	NEC (>30)	tub (>30)	70–90	3	0	(+) NEC	No	Liver (15, NEC)	Death (22)
5	Shin et al.^17)^	2017	32	M	Ascending	tub2	RHC	NEC (>30)	por (>30)	N/D	3	2b (N/D)	N/D	FOLFOX	No	Alive (4)
6	Tagai et al.^18)^	2017	36	F	Ascending	tub	RHC	NEC (>30)	tub (>30)	>70	1b	0	(+) NEC	No	Liver (5, NEC)	Death (20)
7	Guadagno et al.^19)^	2019	65	M	N/D	HGD, UC	Total proctocolectomy	NEC (>30)	tub1 (>30)	70	2	1a (NEC)	N/D	N/D	N/D	N/D
8	Carboni et al.^20)^	2019	54	M	Cecum	muc	RHC + peritonectomy + HIPEC	NEC (60)	muc (40)	80	4b	0	(+) N/D	No	Abdominal (12, unknown)	Death (24)
9	Lim et al.^21)^	2020	48	F	Anal canal	NEC	Local excision	NEC (>30)	tub (>30)	80	1	0	(+) NEC	Cisplatin + etoposide → RT	Liver (9, unknown)	Death (10)
10	Al Buthi et al.^22)^	2022	57	F	Transverse	MiNEN, UC	Total colectomy	NEC (60)	sig (40)	75	3	2b (N/D)	N/D	No	Unknown	Death (6)
11	Tanaka et al.^23)^	2025	72	F	Descending	por	Descending colectomy	NEC (70)	muc (30)	90	4a	1b (muc)	(+) NEC	Cisplatin + irinotecan	No	Alive (24)
12	Fujimura et al.^24)^	2025	75	M	Rectum	Not performed	EMR → anterior resection	NEC (>30)	tub2 (>30)	30	3	1a (NEC)	(+) NEC	No	No	Alive (36)
13	Present case	2026	66	M	Cecum	tub2	Ileocecal resection	NEC (60)	tub2 (40)	50–60	3	1a (tub2)	(+) NEC + tub2	Carboplatin + etoposide	No	Death (24)

Reported cases were identified through a PubMed search of English-language literature published between 2010 and 2025, following the introduction of the term “mixed adenoneuroendocrine carcinoma (MANEC)” in the 2010 WHO classification. The search was performed using combinations of the keywords “MiNEN,” “MANEC,” “colon,” “rectal,” and “colorectal.” Only localized colorectal collision-type MiNENs composed of adenocarcinoma and NEC were included.

EMR, endoscopic mucosal resection; F, female; HGD, high-grade dysplasia; HIPEC, hyperthermic intraperitoneal chemotherapy; M, male; MiNEN, mixed neuroendocrine–non-neuroendocrine neoplasm; muc, mucinous adenocarcinoma; N, pathological N classification according to the TNM Classification of Malignant Tumors, 8th Edition; N/D, not described; NE, neuroendocrine component; NEC, neuroendocrine carcinoma; por, poorly differentiated adenocarcinoma; RHC, right hemicolectomy; RT, radiotherapy; sig, signet ring cell carcinoma; T, pathological T classification according to the TNM Classification of Malignant Tumors, 8th Edition; tub(1/2), (well/moderately) differentiated adenocarcinoma; UC, ulcerative colitis

Several retrospective studies have suggested that postoperative adjuvant chemotherapy may improve outcomes in patients with NECs and MiNENs.^25–29)^ Current guidelines recommend considering adjuvant chemotherapy after curative resection, and platinum-based regimens, including cisplatin or carboplatin combined with etoposide, are generally regarded as the standard treatment for NEC.^9,10)^ However, the optimal adjuvant regimen for MiNEN remains undefined because the relative biological significance of each component may differ. Importantly, lymph node and distant metastases may exhibit distinct metastatic patterns; the major pathological component in regional lymph nodes may reflect the relative proportion of each component within the primary tumor, whereas distant metastases are often dominated by the NEC component.^30)^ Accordingly, lymph node metastasis may not reliably represent the most biologically aggressive component. Furthermore, previous studies have suggested that NECs with high proliferative activity, particularly Ki-67 indices exceeding 55%, are associated with an especially poor prognosis.^5,6)^ In the present case, despite lymph node metastasis consisting solely of adenocarcinoma, the NEC component accounted for 60% of the primary tumor and exhibited several adverse pathological features, including small-cell morphology, lymphovascular invasion, deep mesenteric invasion, and a Ki-67 index of approximately 50%–60%, raising concern for potential systemic dissemination. Consequently, NEC-oriented adjuvant chemotherapy was administered after curative resection, and no recurrence was observed during follow-up. Carboplatin was selected because the patient had chronic kidney disease and multiple comorbidities, making cisplatin-based treatment less suitable. Although the contribution of adjuvant chemotherapy cannot be determined from a single case, this case highlights the importance of comprehensive pathological assessment of the resected specimen for postoperative therapeutic decision-making in colorectal MiNEN.

## CONCLUSIONS

Colorectal MiNEN poses substantial diagnostic challenges because biopsy specimens may not adequately represent both tumor components. Comprehensive pathological assessment of the resected specimen is important for identifying the aggressive component most likely to influence prognosis and for optimizing postoperative therapeutic strategy.
